# Mechanical micronization of lipoaspirates for the treatment of hypertrophic scars

**DOI:** 10.1186/s13287-019-1140-1

**Published:** 2019-01-24

**Authors:** Jing Wang, Yunjun Liao, Jing Xia, Zijue Wang, Xiaopei Mo, Jingwei Feng, Yunfan He, Xihang Chen, Ye Li, Feng Lu, Junrong Cai

**Affiliations:** Department of Plastic and Reconstructive Surgery, Nanfang Hospital, Southern Medical University, Guangzhou, 510515 China

**Keywords:** Hypertrophic scar, Stromal vascular fraction, Fat grafting, Myofibroblast, Macrophage

## Abstract

**Background:**

Hypertrophic scars cause cosmetic and functional problems for patients, and their treatment remains challenging. Mechanical micronization of adipose tissue can remove adipocytes and concentrate functional cells. Stromal vascular fraction (SVF)-gel is obtained by a series of simple mechanical processes, including shifting between syringes and centrifugation. This study aimed to assess the therapeutic effect of SVF-gel on hypertrophic scars.

**Methods:**

A model of hypertrophic scars was established in rabbit ears. SVF-gel and SVF cells were obtained from rabbit inguinal fat pads and injected into scars. Phosphate-buffered saline (PBS) was used as a control. Scars were structurally characterized by histologic and immunohistochemical analyses. Expression of inflammatory and fibrogenic genes was evaluated.

**Results:**

Hypertrophic scars became less visible and softer following injection of SVF-gel or SVF cells. Dermal thickness was significantly lower in the groups treated with SVF-gel and SVF cells than in the PBS-treated group. Treatment with SVF-gel restored subcutaneous fat tissue in scars, while treatment with SVF cells and PBS did not. Injection of SVF-gel and SVF cells reduced macrophage infiltration in the dermal layer and decreased mRNA expression of interleukin-6 and monocyte chemoattractant protein-1. In addition, the level of myofibroblasts and collagen deposition were reduced in the groups treated with SVF-gel and SVF cells.

**Conclusions:**

SVF-gel has therapeutic effects on hypertrophic scars. Injection of SVF-gel into hypertrophic scars restores subcutaneous fat tissue and reduces the levels of macrophages and myofibroblasts; thus, it decreased the dermal thickness of the scar.

## Introduction

Hypertrophic scars are thickened and raised scars that develop due to abnormal collagen deposition and remodeling following injury of dermal tissue [1]. Hypertrophic scars can cause cosmetic and functional problems for patients. Current therapeutic options for hypertrophic scars include corticosteroid treatment, laser therapy, and surgical removal [2]. However, these treatments fail to effectively eliminate excessive scar tissue and regenerate healthy dermal tissue. Hence, there is no gold standard therapy, and treatment of hypertrophic scars remains challenging.

Fat tissue-derived stem cell products, such as stromal vascular fraction (SVF) and adipose-derived stem cells (ASCs), improve the elasticity of scar tissue by modulating collagen deposition and stimulating blood vessel regeneration, indicating they have therapeutic potential for scars [3–5]. ASCs inhibit the proliferation of scar tissue by reducing the activities of myofibroblasts and mast cells, thereby preventing stimulation of fibroblasts by transforming growth factor (TGF)-β1 [6]. Co-culture of ASCs and fibroblasts reduces the proliferative capacity of fibroblasts and decreases expression of TGF-β1, phosphorylated Smad2/3, and type I/III collagen, which function in the TGF-β1/Smad signaling pathway [7]. ASCs block the TGF-β1-Smad3 interaction and reverse the TGF-β1 and Smad3 signaling pathway via crosstalk with other signaling pathways, thereby preventing pathologic scar formation. However, adipose tissue must be digested with collagenase for 30–60 min to obtain ASCs, which increases the risk of preparations being contaminated by exogenous substances and unwanted biological materials [8]. Moreover, adherent culture and purification of ASCs require specialized laboratory equipment and take days to weeks. Consequently, the therapeutic application of ASCs is limited.

Several mechanical processing procedures, including centrifugation, mechanical chopping, shredding, pureeing, and mincing, were developed to obtain ASCs from adipose tissue without collagenase-mediated digestion [9–11]. These methods are thought to condense tissue and ASCs by mechanically disrupting mature adipocytes and their oil-containing vesicles. Moreover, these preparations, which contain a high density of ASCs, show considerable therapeutic potential as a regenerative medicine [12]. We previously reported a mechanical processing method that removes most lipids and fluid from lipoaspirates and leaves only SVF cells and fractionated extracellular matrix (ECM) [13]. This product is called SVF-gel. SVF-gel is prepared from fat aspirates by centrifugation and shifting between syringes. This preparation has a smooth texture and can be injected through a fine (27-gauge) needle. SVF-gel is enriched in ASCs, vascular endothelial cells (ECs), and native adipose ECM.

SVF-gel contains many cells and can be injected though a fine needle. Consequently, SVF-gel is a potential regenerative treatment for hypertrophic scars. Herein, we aimed to compare the therapeutic effects of SVF-gel and SVF cells on hypertrophic scars.

## Materials and methods

### Animals

This study was approved by the Animal Experimentation Ethics Committee of Nanfang Hospital, Southern Medical School, China. All animal treatments were carried out in accordance with the Guidelines on Care and Use of Laboratory Animals issued by the Chinese Council on Animal Research and the Guidelines of Animal Care. New Zealand white rabbits were purchased from the Experimental Animal Center of Southern Medical School and had an initial body weight of 2.5 ± 0.2 kg. Animals were housed in a regulated environment (22 ± 2 °C) with a 12-h light/dark cycle. All animals were fed according to the specific pathogen-free animal criteria.

### Preparation of SVF-gel

The procedure used to produce rabbit SVF-gel was modified from a previous report that prepared human SVF-gel [13]. Rabbits were intravenously anesthetized with 30 mg/kg sodium pentobarbital. The right inguinal area was shaved and cleaned under aseptic operation conditions, and then, the inguinal fat pad was exposed by making a 3-cm incision. Thereafter, 5 mL of pure fat was harvested, cleared with normal saline, and dried with gauze. The fat pad was cut into tiny pieces by continuous fine mincing with surgical scissors for 2 min and then mechanically emulsified by transfer between syringes at a rate of 10 mL/s for 1 min. The morcellated tissue was transferred to a 10-mL tube and centrifuged at 2000*g* for 3 min. The substance below the oil layer was defined as SVF-gel and collected for further use.

### Isolation of SVF cells and flow cytometry

SVF cells were isolated from fat pads and SVF-gel by collagenase digestion. Briefly, SVF-gel and fat pads were digested with phosphate-buffered saline (PBS) containing 0.075% collagenase for 30 min on a shaker at 37 °C. Mature adipocytes and connective tissue were removed by centrifugation at 800*g* for 5 min. The cell pellet was resuspended and filtered through a 100-μm mesh. The number of SVF cells was counted. SVF cells prepared from SVF-gel were used for flow cytometric analysis and culture of ASCs, while SVF cells prepared from fat pads were used to treat scars.

SVF cells prepared from SVF-gel were characterized by fluorescence-activated cell sorting analysis. The cells were neutralized, washed, filtered, counted, and labeled with the following dyes, antibodies, and corresponding isotype controls for 30 min: 7-AAD (BD Pharmingen, Franklin Lakes, NJ, USA), anti-CD31-Pacific Blue (BioLegend, San Diego, CA, USA), anti-CD34-PE (BD Pharmingen), and anti-CD45-FITC (Thermo Fisher, Waltham, MA, USA). SVF cells were analyzed using an LSR II flow cytometer (Becton Dickinson, San Jose, CA, USA) and counted using an AMQAF 1000 device (Thermo Fisher Scientific, Fremont, CA, USA). Gates were set for isotype controls to exclude > 99.9% of non-specifically stained cells for classification of the cell populations.

### Differentiation of ASCs

SVF cells obtained from SVF-gel were cultured overnight at 37 °C in 5% CO_2_ in control medium (Dulbecco’s modified Eagle’s medium containing 10% fetal bovine serum, 100 U/mL penicillin, and 100 mg/mL streptomycin). The resulting cell population was cultured for 3–5 days until fully confluent. ASCs were cultured and expanded in control medium. Cells from passages 3–5 were used. In vitro multilineage differentiation of ASCs was induced in control medium supplemented with one of the three formulae described below as described previously [14]. ASCs were stained with Oil Red O, Alizarin red, and Alcian blue to identify fat, bone, and cartilage cells, respectively.

### Scanning electron microscopy (SEM)

Normal fat tissue and SVF-gel samples were fixed with 2% glutaraldehyde in 0.1 M phosphate buffer, post-fixed in 1% osmium tetroxide in the same buffer for 1 h. Then, it was dehydrated in increasing concentrations of acetone, critical-point dried, fixed to stubs with colloidal silver, sputtered with gold using a MED 010 coater, and examined under an S-3000 N scanning electron microscope (HITACHI Company, Japan).

### Rabbit ear model of hypertrophic scars

The rabbit ear model of hypertrophic scars was established according to a previously described procedure [15]. In brief, rabbits were intravenously anesthetized with 30 mg/kg sodium pentobarbital. A rectangular wound (5.5 cm long and 1.5 cm wide) was created down to the bare cartilage on the ventral surface of each ear along the long axis under aseptic conditions. The epidermis, dermis, and perichondrium were completely removed, and the wound was covered with sterile gauze for 1 day. Thereafter, the rabbits were provided conventional anti-inflammatory treatment. Four weeks after the operation, the healed wound surface exhibited an obvious protrusion, indicative of hypertrophic scar formation.

Forty-five rabbits were divided into three groups (SVF-gel, SVF cells, and PBS), which were matched in terms of age and gender. Four weeks after the operation, 1 × 10^5^ SVF cells suspended in 1 mL of PBS or 1 mL of SVF-gel was injected into the scar. PBS was used as a control.

### General observation and histological analysis

General observations of hypertrophic scars, including size, color, texture, and thickness, were recorded by photographic imaging at the designated time points.

Full-thickness biopsies of the scar and surrounding tissue were obtained at 1, 4, and 12 weeks after treatment. Samples were embedded in paraffin, sectioned, and stained with hematoxylin and eosin (H&E) and Masson’s trichrome (MT). Staining was performed according to a standard protocol. Sections were examined underneath an Olympus BX51 microscope and evaluated. Dermal thickness and collagen density were measured by analyzing images using ImageJ.

Immunohistochemical analysis was performed following the standard procedures. Briefly, paraffin-embedded tissue sections were incubated with a rabbit anti-α-smooth muscle actin (α-SMA; 1:60; Abcam, Cambridge, MA, USA) followed by a secondary antibody. Signals were observed using an avidin-biotin-horseradish peroxidase detection system. Slides were examined under an Olympus BX51 microscope. To quantitate myofibroblasts, two of the authors counted the number of α-SMA-positive cells in at least five fields per sample in a double-blinded fashion.

The following primary antibodies were used for immunofluorescence staining: rabbit anti-mouse CD206 (1:300; Abcam) and guinea-pig anti-mouse perilipin (1:200; Progen, Heidelberg, Germany). The following secondary antibodies were used for co-staining: donkey anti-rat IgG-555 (1:200; Abcam) and goat anti-chicken IgY-488 (1:200; Thermo Fisher, Cambridge, MA, USA). Nuclei were stained with DAPI (1:200; Sigma, St. Louis, MO, USA). Images were acquired and analyzed using a confocal laser scanning microscope (C1Si; Nikon, Tokyo, Japan).

### Quantitative reverse transcription PCR

Skin and fat tissue was rapidly excised separately, immediately frozen in liquid nitrogen, and stored at − 80 °C. Total RNA was extracted from 50 mg of frozen tissue using an RNeasy Lipid Tissue Mini Kit (Qiagen, Hilden, Germany) according to the manufacturer’s instructions. cDNA was amplified in 40 cycles using a QuantiTect Reverse Transcription Kit (Qiagen) and a Rotor-Gene 3000 Real-Time PCR Detection System (Corbett Research, Sydney, Australia). GAPDH was used as a reference gene. Expression levels were calculated using the 2-ΔΔCt method. The following primers were used: monocyte chemoattractant protein (MCP)-1, forward 5′-GCAAGATGATCCCAATGAGT-3′ and reverse 5′-TAGCTTCAGATTTACGGGTC-3′; interleukin (IL)-6, forward 5′-CCTGTCTATACCACTTCACA-3′ and reverse 5′-TGCATCATCGTTGTTCATAC-3′; collagen I (Col-1), forward 5′-GAGCAACATGTGGAACTCTA-3′ and reverse 5′-TGAATCGAAAGCCCTGTATT-3′; and GAPDH, forward 5′-GGCCTCCAAGGAGTAAGAAA-3′ and reverse 5′-GCCCCTCCTGTTATTATGG-3′.

### Statistical analysis

All statistical analyses were performed using SPSS 22.0 software, and all data are expressed as mean ± SD. Data were compared between three groups using a one-way analysis of variance and the Kruskal-Wallis test. Data were compared between two groups using the least significant difference method and the Mann-Whitney *U* test. *p* < 0.05 was considered statistically significant.

## Results

### Cellular components of SVF-gel

**Fig. 1:**
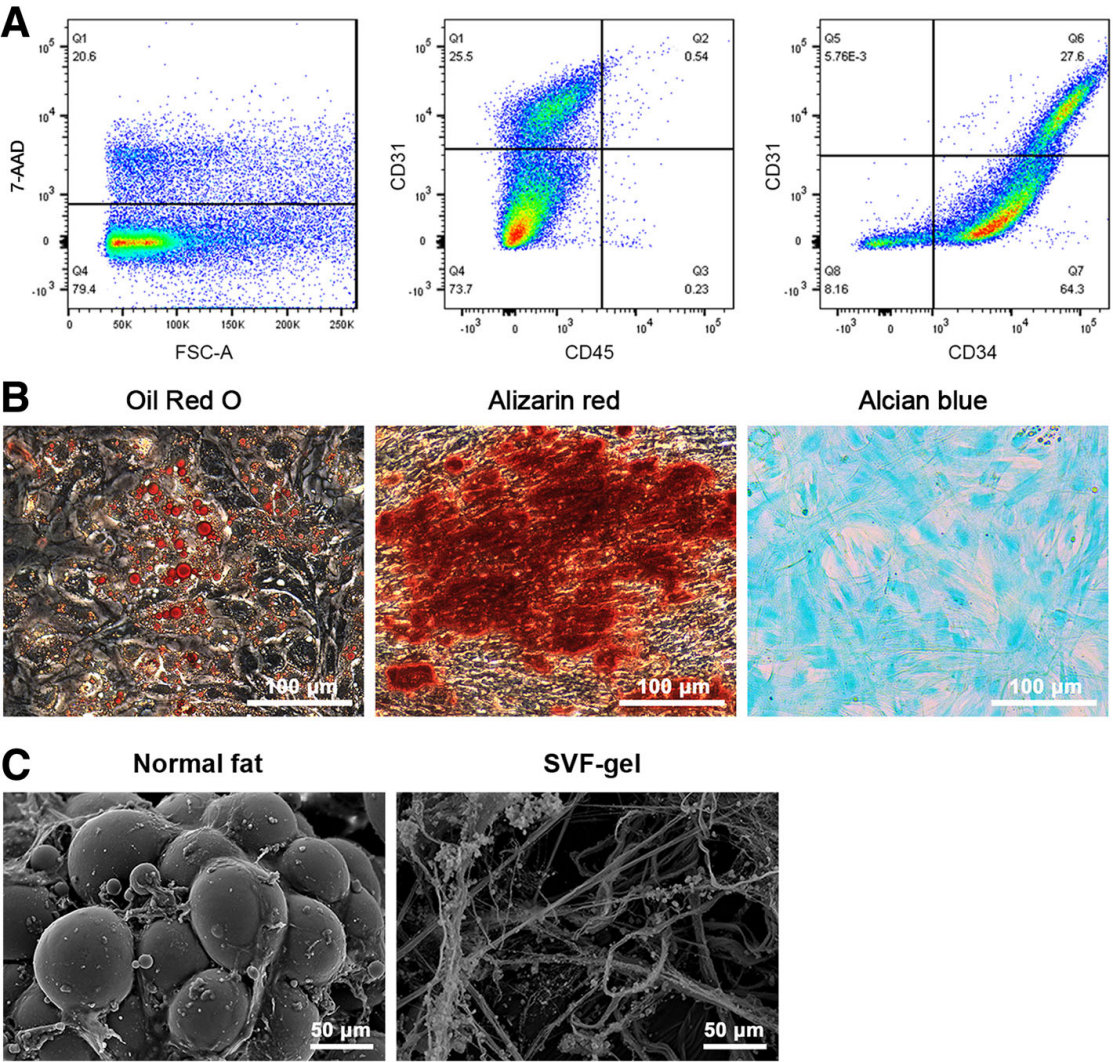
Characterization of rabbit SVF-gel. **a** Flow cytometry showed that 20% of SVF cells were 7-AAD-positive. There were two main cell populations: CD34+/CD31−/CD45− ASCs and CD34+/CD31+/CD45− ECs. **b** Adipogenic, osteogenic, and chondrogenic differentiation of ASCs was determined by staining intracellular lipid droplets with Oil Red O (left), staining matrix mineralization with Alizarin red (middle), and staining glycosaminoglycans with Alcian blue (right), respectively. Scale bars = 100 μm. **c** Scanning electron microscopy showed that normal rabbit fat tissue had an intact structure with adipocytes attached to extracellular fibers, while SVF-gel contained no adipocytes within it

The cellular components of SVF-gel were characterized by flow cytometry (Fig. 1a). Staining of dead cells with 7-AAD demonstrated that 80% of SVF cells were alive in SVF-gel. Two main cell populations were obtained from SVF-gel: CD34+/CD31−/CD45− ASCs, which accounted for 64% of SVF cells, and CD34+/CD31+/CD45− ECs, which accounted for 28% of SVF cells. To verify their multipotency, ASCs were incubated in media that induce adipogenic, osteogenic, and chondrogenic differentiation. Staining of intracellular lipid droplets with Oil Red O, staining of matrix mineralization with Alizarin red, and staining of glycosaminoglycans with Alcian blue confirmed that ASCs had the capacity to undergo adipogenic, osteogenic, and chondrogenic differentiation, respectively (Fig. 1b). Scanning electron microscopy was performed to examine changes in the extracellular matrix structure after the mechanical process (Fig. 1c). The normal fat had an intact structure with adipocytes attached to extracellular fibers. However, no adipocytes existed within the SVF-gel.

### Successful establishment of a rabbit ear model of hypertrophic scars

**Fig. 2:**
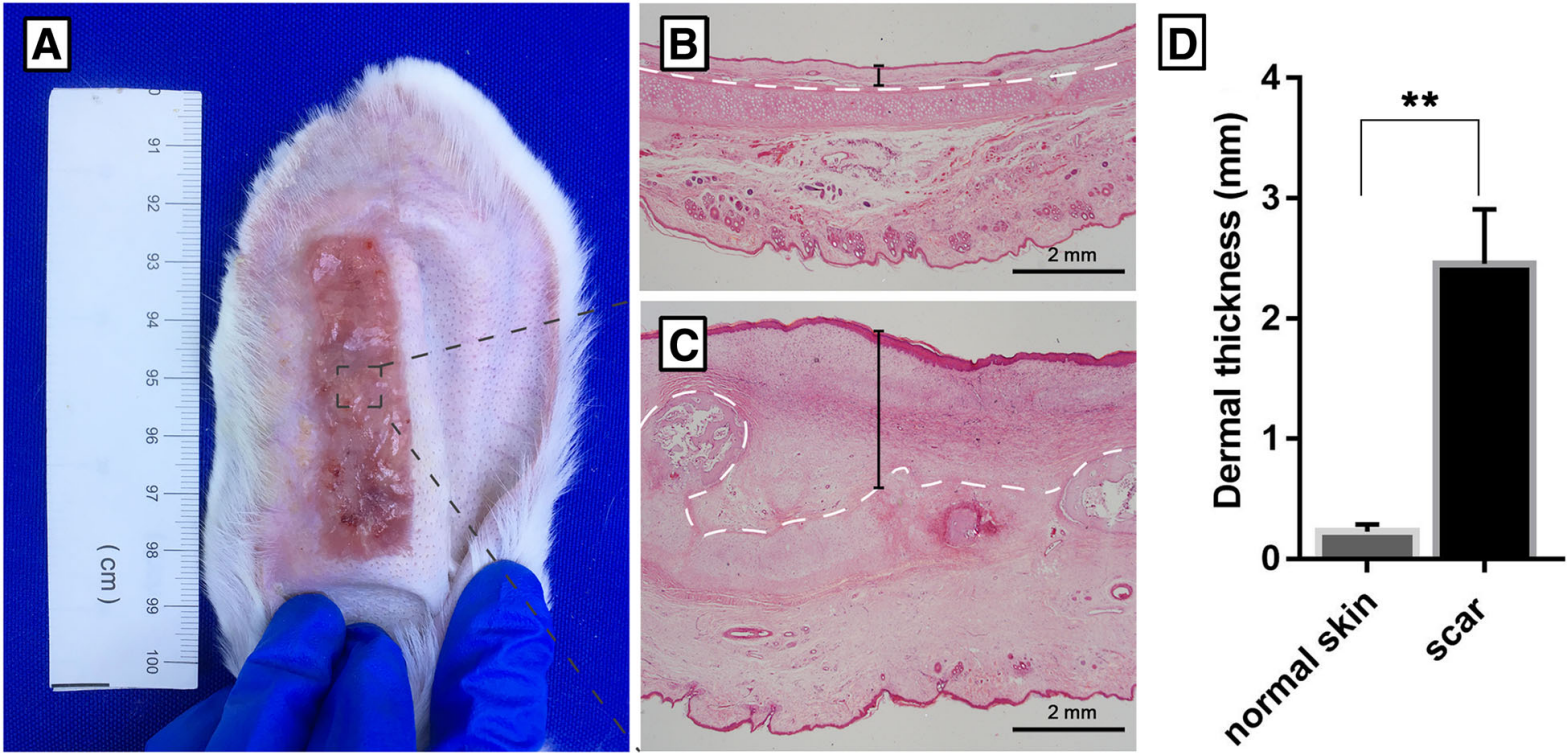
Establishment of a rabbit ear model of hypertrophic scars. **a** A rectangular hypertrophic scar (5.5 cm long and 1.5 cm wide) developed at 4 weeks after the operation. **b** The dermal layer (the area above the white dotted lines) was thin in normal skin on a rabbit ear. Scale bar = 2 mm. **c** Irregular hypertrophy was observed in the dermal layer of the scar. The dermal thickness (shown in the black lines) was dramatically increased. Scale bar = 2 mm. **d** The dermal thickness was measured and significantly increased in the scar (normal skin 0.23 ± 0.02 mm; scar 2.45 ± 0.19 mm; **p < 0.01)

A rectangular wound was created in the ear of a rabbit. Four weeks later, re-epithelization was complete and a stiff and visibly raised scar had formed (Fig. 2a). Compared with normal skin on the ear (Fig. 2b), the scar exhibited extensive collagen deposition and a decreased level of subcutaneous adipose tissue (Fig. 2c). Dermal thickness was significantly higher in the scar than in normal skin (normal skin 0.23 ± 0.02 mm; scar 2.45 ± 0.19 mm; *p* < 0.01; Fig. 2d).

### SVF-gel and SVF cells improve hypertrophic scars

**Fig. 3:**
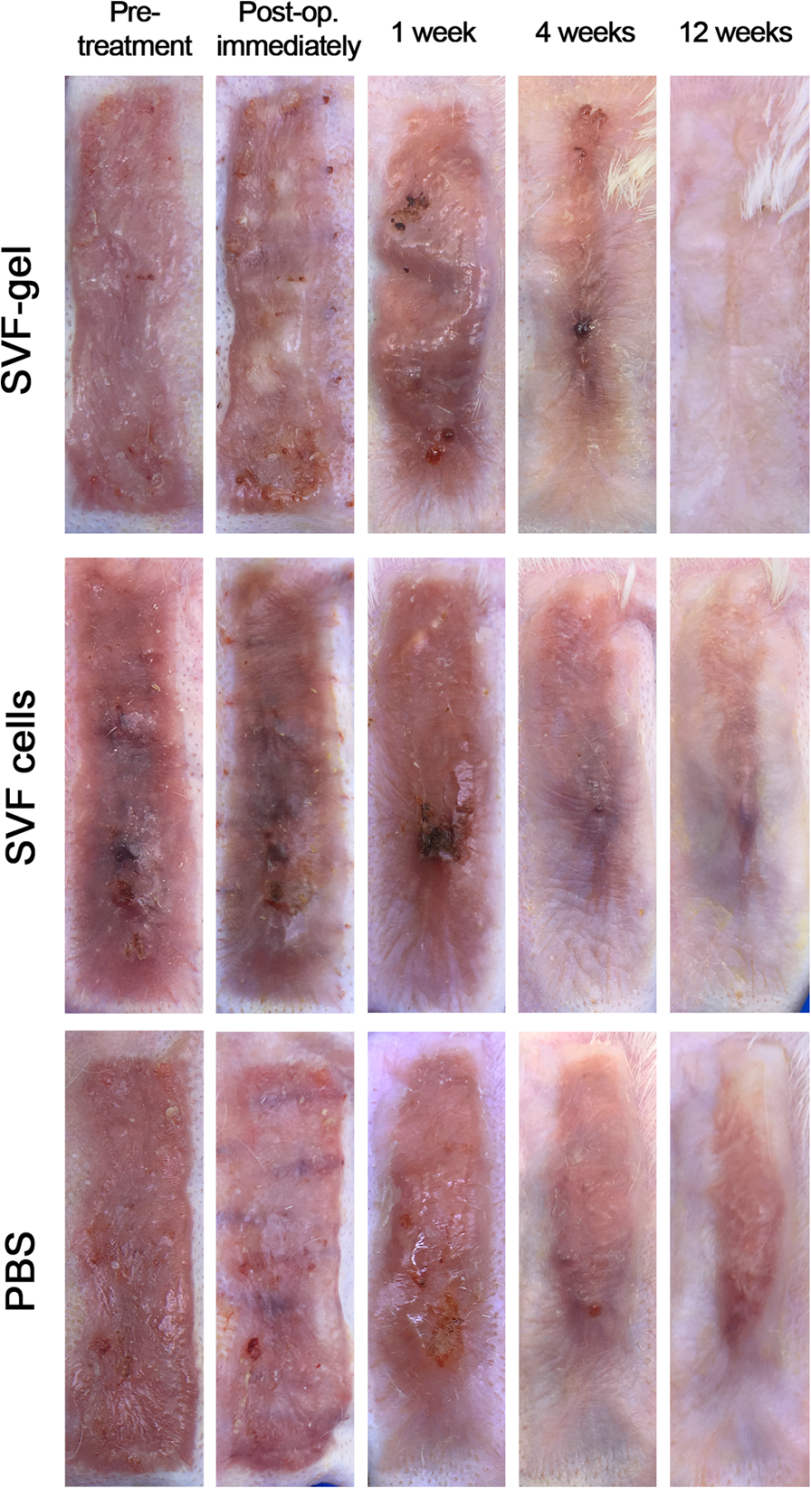
Gross observation of hypertrophic scars after treatment with PBS, SVF cells, and SVF-gel. (Upper) Scars were barely noticeable after 12 weeks in the SVF-gel-treated group. (Middle) Scars became less visible and softer in the SVF cell-treated group. (Lower) A stiff and visibly raised scar persisted for more than 12 weeks in the PBS-treated group

The same size scars were created among these three groups. In the SVF-gel group, the injected SVF-gel can be visualized beneath the scar immediately and 1 week after injection. Four weeks post-injection, the injected SVF-gel could not be seen and the scar became much softer and had much smaller size, compared with the other two groups. A stiff and visibly raised scar persisted for more than 12 weeks in the control group. By contrast, scars became markedly less visible and softer in the SVF-gel- and SVF cell-treated groups. Surprisingly, scars were barely noticeable after 12 weeks in the SVF-gel-treated group (Fig. 3).

### SVF-gel restores normal cutaneous structures in hypertrophic scars

**Fig. 4:**
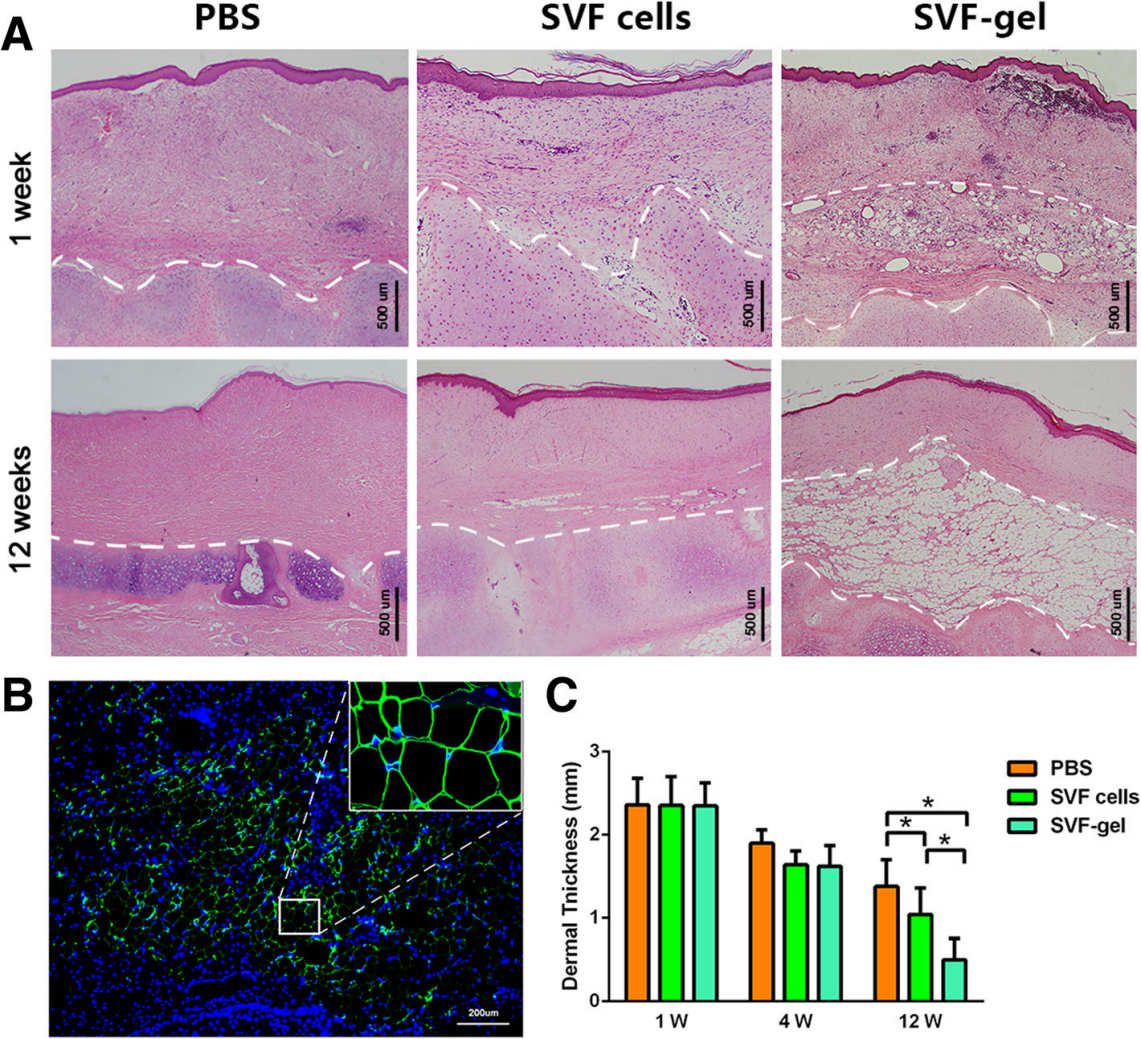
SVF-gel restores normal cutaneous structures in hypertrophic scars. **a** H&E staining showed that skin had a normal structure with a complete epidermis, a thinner dermal layer, and substantial subcutaneous fat tissue in the SVF-gel-treated group. However, little subcutaneous fat tissue was observed in the PBS- and SVF cell-treated groups. Scale bars = 500 μm. **b** Restoration of subcutaneous fat tissue in the SVF-gel-treated group was confirmed by immunofluorescence staining of perilipin. Scale bar = 200 μm. **c** At week 12, dermal thickness was reduced most by treatment with SVF-gel (SVF-gel, 0.50 ± 0.25 mm; PBS, 1.38 ± 0.32 mm; *p < 0.05) and was also decreased by treatment with SVF cells (SVF cells, 1.04 ± 0.32 mm, *p < 0.05)

At 1 week post-treatment, transplanted SVF-gel was observed beneath the dermal layer and small oil cysts were detected. Inflammatory cell infiltration was high in all three groups at this time point. At 12 weeks post-treatment, skin in the SVF-gel-treated group had a normal structure, with a complete epidermis, a thinner dermal layer, and substantial subcutaneous fat tissue. However, little subcutaneous fat tissue was observed in the PBS- and SVF cell-treated groups (Fig. 4a). Restoration of subcutaneous fat tissue in the SVF-gel-treated group was confirmed by immunofluorescence staining of perilipin (Fig. 4b). Moreover, dermal thickness was gradually decreased in the process of time in all three groups. No significant difference among the three groups was observed at weeks 1 and 4. At week 12, it is reduced most in the SVF-gel-treated group (SVF-gel, 0.50 ± 0.25 mm; PBS, 1.38 ± 0.32 mm; *p* < 0.05) and was also significantly reduced in the SVF cell-treated group (SVF cells, 1.04 ± 0.32 mm; *p* < 0.05; Fig. 4c).

### SVF-gel decreases macrophage infiltration in hypertrophic scars

**Fig. 5:**
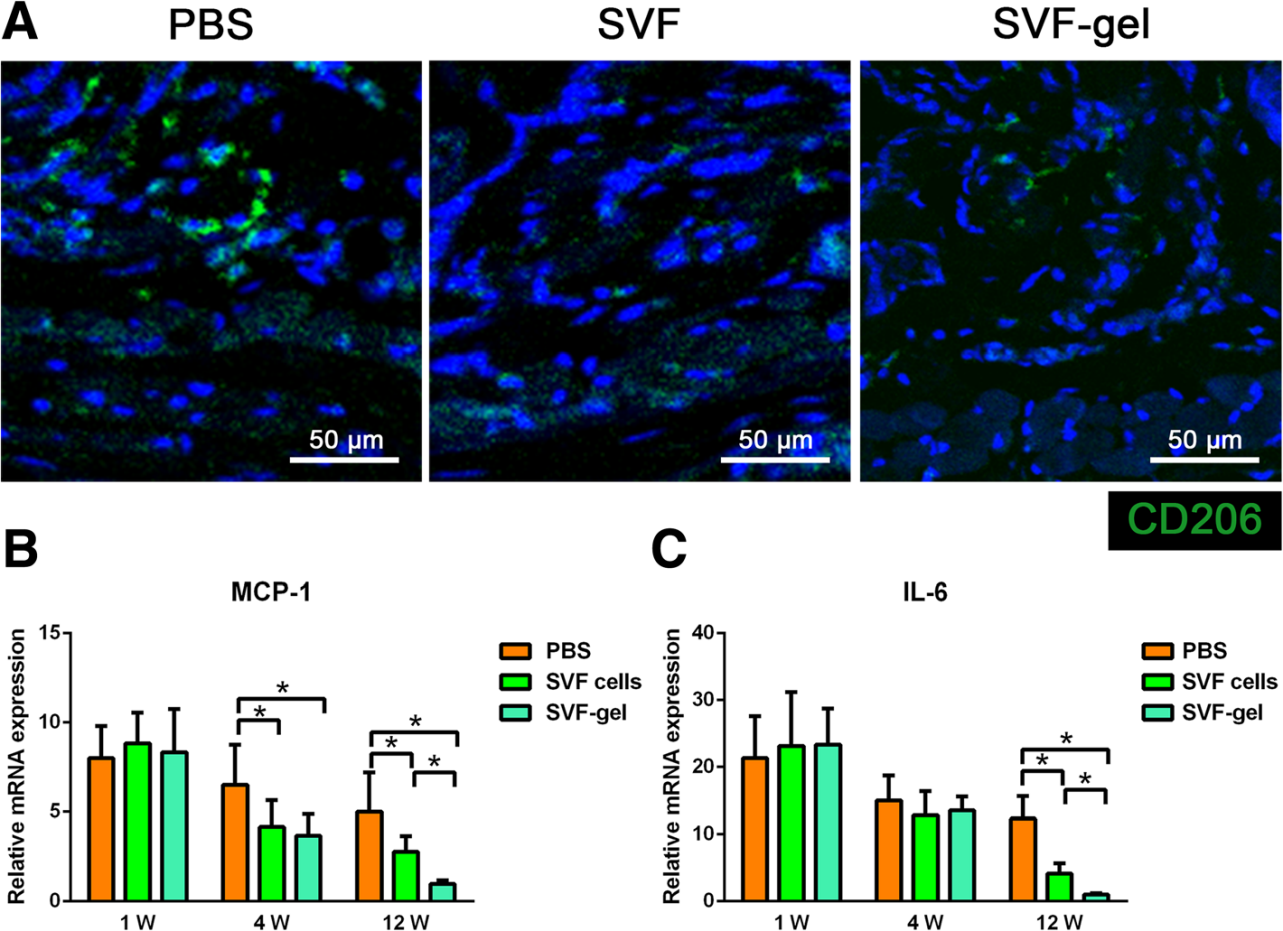
SVF-gel decreases macrophage infiltration in hypertrophic scars. **a** Immunofluorescence staining of the 12-week samples showed that treatment with SVF-gel and SVF cells decreased infiltration of macrophages (CD206+) in the dermal layer. **b** Treatment with SVF-gel and SVF cells suppressed mRNA expression of MCP-1 (*p < 0.05). **c** Treatment with SVF-gel and SVF cells suppressed mRNA expression of IL-6 (*p < 0.05)

Immunofluorescence staining showed that infiltration of macrophages (CD206+) in the dermal layer was decreased in the SVF-gel- and SVF cell-treated groups (Fig. 5a). We further evaluated the expression of the inflammatory cytokines MCP-1 and IL-6. No significant difference of MCP-1 mRNA expression was observed among the three groups at week 1. At weeks 4 and 12, mRNA expression of MCP-1 was significantly lower in the SVF-gel- and SVF cell-treated groups than in the PBS-treated group (*p* < 0.05, respectively, Fig. 5b). As for the mRNA expression of IL-6, no significant difference was observed among the three groups at week 1. At week 12, mRNA expression of IL-6 was significantly lower in the SVF-gel- and SVF cell-treated groups than in the PBS-treated group (*p* < 0.05, Fig. 5c).

### SVF-gel reduced the fibrotic tendency in hypertrophic scars

**Fig. 6:**
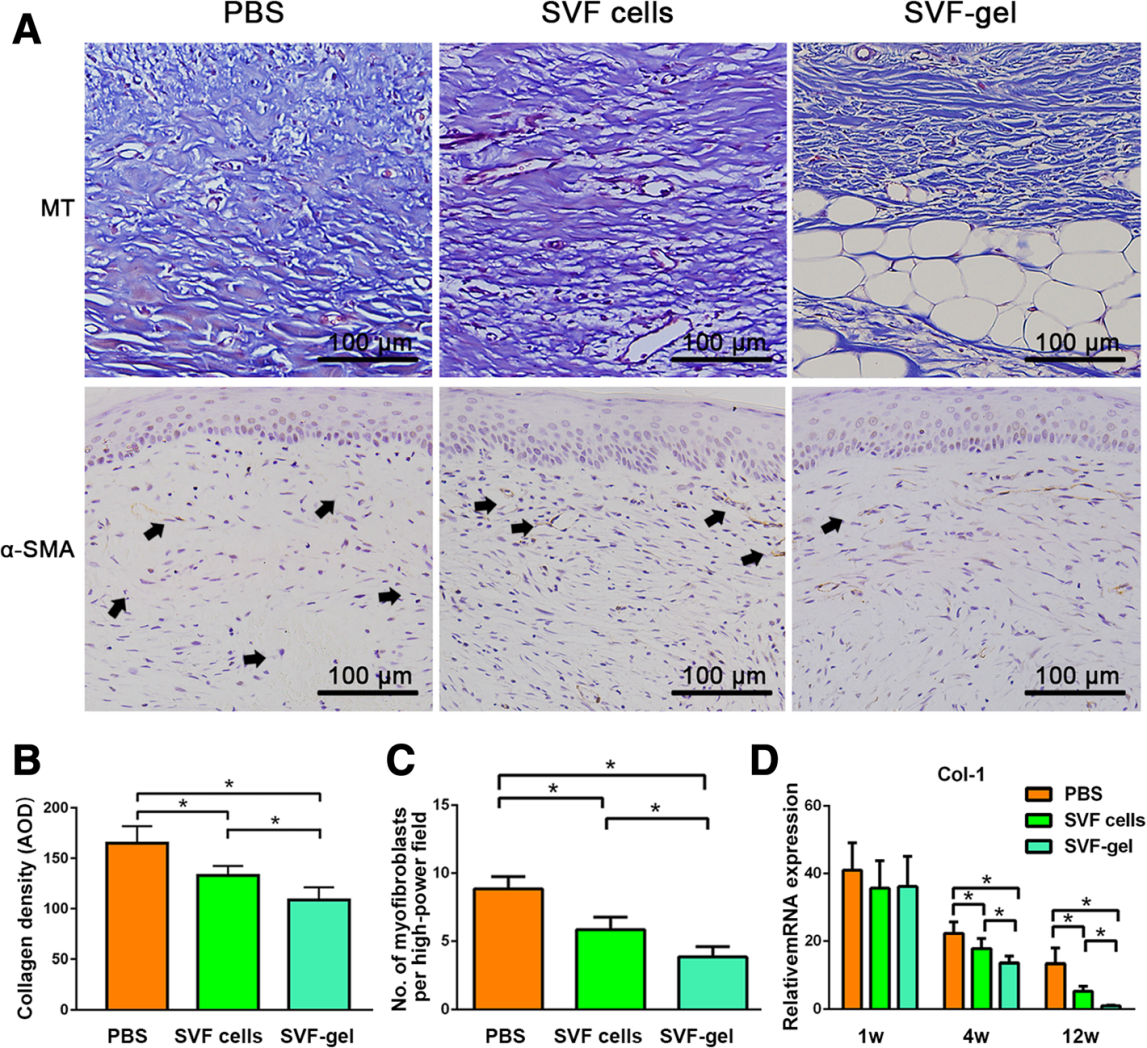
SVF-gel reduces the fibrotic tendency in hypertrophic scars. **a** (Upper) MT staining of the 12-week samples showed that collagen fibers were dense and irregularly arranged in the PBS-treated group. Collagen deposition was reduced and collagen fibers were well-arranged in the SVF-gel- and SVF cell-treated groups. **a** (Lower) Immunostaining of α-SMA of the 12-week samples showed that myofibroblasts (black arrows) were present in the dermal layer. **b** At week 12, quantification of collagen density showed that PBS-treated group had the highest collagen density, while collagen density was significantly reduced in SVF-gel group (*p* < 0.05). **c** Quantification of myofibroblasts in scars showed that myofibroblast infiltration was lowest in the SVF-gel-treated group at week 12 (*p < 0.05). **d** Quantitative reverse transcription PCR of Col-1 expression in scars in the three groups. No significant difference was observed among the three groups at week 1. Col-1 expression was lower in the SVF-gel-treated group than in the PBS- and SVF cell-treated groups since week 4 (*p < 0.05)

MT staining was performed to display the collagen fibrosis. At 12 weeks post-treatment, collagen fibers were dense and irregularly arranged in the PBS-treated group. Conversely, collagen fibers in the SVF-gel group had dramatically reduced, the blue staining had faded, the fiber bundles had become thinner, and the alignment had loosened (Fig. 6a, upper). At week 12, the quantification of collagen density showed that PBS-treated group had the highest collagen density, while collagen density was significantly reduced in SVF-gel group (*p* < 0.05, Fig. 6b).

In scar tissue, spindle-shaped cells positive for α-SMA are regarded as myofibroblasts, which play a key role in fibrosis during hypertrophic scarring. Immunostaining of α-SMA showed that myofibroblasts (black arrows) were present in the dermal layer (Fig. 6a, lower). At week 12, the number of myofibroblasts in the dermis of scars was significantly reduced in the SVF-gel-treated group and was highest in the PBS-treated group (Fig. 6c). The number of myofibroblasts significantly differed between the three groups (*s* < 0.05).

We further analyzed Col-1 expression in scars (Fig. 6d). No significant difference was observed among the three groups at week 1. At weeks 4 and 12, Col-1 expression was significantly higher in the PBS-treated group than in the SVF-gel- and SVF cell-treated groups (*p* < 0.05). In addition, Col-1 expression was significantly lower in the SVF-gel-treated group than in the SVF cell-treated group (*p* < 0.05).

## Discussion

Stem cell therapy has great potential for tissue regeneration and wound healing [16–18]. SVF cells and ASCs isolated from adipose tissue are frequently used for tissue regeneration [19–21] and have therapeutic effects on scars [4, 6]. However, adipose tissue must be digested with collagenase to obtain these cells, which might increase the risk of biological contamination and requires specialized laboratory equipment. Consequently, therapeutic alternatives are needed.

ASCs can also be concentrated via mechanical processing of fat tissue. SVF-gel is produced by mechanical processing of adipose tissue, which involves shifting between syringes and centrifugation [13]. We previously reported that the density of ASCs is much higher in SVF-gel than in unprocessed lipoaspirates [13]. In the current study, we produced SVF-gel from rabbit adipose tissue. Flow cytometry demonstrated that ~ 64% of SVF cells in SVF-gel were CD45−/CD31−/CD34+ ASCs. Moreover, these cells could proliferate and differentiate into various lineages. During the process used to obtain SVF-gel, centrifugation generates high-density fat layers that contain many viable adipocytes and SVF cells. This high-density fat is emulsified and centrifuged to remove adipocytes and thereby further increase the density of stem cells, which remain viable and functional. Moreover, SVF-gel is collagenase-free, while enzymes are required to produce SVF cell suspensions. Thus, SVF-gel might be a better adipose tissue-derived product for regenerative medicine.

In this study, a rabbit hypertrophic scar model was applied. This model was proved to parallel hypertrophic scarring in humans [22]. Both SVF-gel and SVF cells elicited therapeutic effects on hypertrophic scars. Although SVF cells were previously reported to improve hypertrophic scars, the underlying mechanism is unknown [4, 5]. ASCs are the key component in either SVF suspension or SVF-gel [13, 23]. It was reported that ASCs suppress production of pro-inflammatory cytokines, such as TNF-α and IL-12, in activated macrophages and induce apoptosis of activated macrophages [24]. ASCs inhibit M1-polarization gene expression in macrophages. Moreover, ASCs inhibit activated macrophages and attenuate inflammation in a mouse model of inflammatory bowel disease. ASCs also inhibit nephritis in a mouse model of glomerulonephritis by converting macrophages to immunoregulatory cells. Mesenchymal stem cells (MSCs) prevent hypertrophic scar formation in rabbit ears by regulating inflammation and secretion of the anti-inflammatory protein TNF-α-stimulated gene/protein 6 [25]. In the present study, we confirmed that SVF cells and SVF-gel attenuated macrophage-mediated inflammation. Both SVF-gel and SVF cells reduced the level of CD206+ macrophages in scars. Moreover, the expression of the inflammatory cytokines IL-6 and MCP-1 was significantly decreased in the SVF-gel- and SVF cell-treated groups.

SVF-gel and SVF cells also decreased the levels of myofibroblasts and Col-1 expression in scars. Synergistic overactivation of macrophages and myofibroblasts was suppressed by the reduction of macrophage-associated inflammation. This may effectively inhibit the fibroinflammatory process. MSCs inhibit transformation of fibroblasts into myofibroblasts in a p53-dependent manner, and this contributes to the anti-scarring effect observed following engraftment of MSCs [26]. Inhibition of excessive secretion of ECM proteins, including Col-1, by myofibroblasts prevents the formation and development of scars.

Our results showed that SVF-gel treatment led to faster recovery and better histologic results than SVF cell treatment. Indeed, the dermal layer was thinnest and subcutaneous fat tissue was restored in the SVF-gel-treated group. Several possible reasons may contribute to the better therapeutic effect of SVF-gel treatment. The SVF-gel had native adipose-derived extracellular matrix to protect the functional cells within it and co-operated with these cells. Stem cells with their niche are likely to work differently with cell suspension upon delivery, thus results in variable efficacy. Our previous study used the cell tracing method to identify the SVF cells in the target area after injection of SVF suspension or SVF-gel. The results showed that little SVF cells from SVF suspension remained in the injection site, while numerous SVF-gel-derived cells existed in the injection site [27]. SVF-gel not only provides physical protection (adipose ECM niche) but also keeps cell-ECM interaction. It was reported that a three-dimensional scaffold could facilitate ASCs to differentiate into capillary component in vivo [28].

Moreover, although treatment with SVF cells also improved scars, it failed to regenerate subcutaneous fat tissue. As the SEM result suggested, no adipocytes existed in the SVF-gel. The subcutaneous fat tissue may have been regenerated by the transferred SVF-gel. Adipocytes have many functions in various tissues besides energy storage, including regulation of metabolism, growth, and immunity. Immune cells and adipocytes may collaborate to promote wound repair by clearing cell debris and fighting infection [29]. An adipocyte-containing biological dressing enhances repair of full-thickness excisional wounds in a murine model [30]. Moreover, a recent study demonstrated that mature adipocytes are present when newly regenerated hair follicles form in sizeable wounds, suggesting that adipocytes contribute to regeneration of cutaneous appendages [31]. The present study demonstrated that SVF-gel restored subcutaneous fat tissue beneath scars, while SVF cells did not. Regenerated adipocytes may help to improve the quality of scars, including skin elasticity and stiffness. Further studies are required to confirm the role of adipocytes in the improvement of scars.

This study reports the effects of SVF-gel on hypertrophic scars. Limited data have been published regarding the therapeutic effects of mechanically micronized fat tissue. Further investigations are required to elucidate the molecular mechanisms underlying the regenerative effect of SVF-gel on hypertrophic scars.

## Conclusions

Our study showed that SVF-gel had great therapeutic effects on hypertrophic scars. Injection of SVF-gel into hypertrophic scars reduced the levels of macrophages and myofibroblasts among the scars and restored subcutaneous fat tissue; thus, it decreased the dermal thickness of the scar.

## Abbreviations

ASC: Adipose-derived stem cell
EC: Endothelial cell
ECM: Extracellular matrix
H&E: Hematoxylin and eosin
IL: Interleukin
MCP: Monocyte chemoattractant protein
MSC: Mesenchymal stem cell
MT: Masson’s trichrome
PBS: Phosphate-buffered saline
SVF: Stromal vascular fraction
TGF: Transforming growth factor
α-SMA: α-Smooth muscle actin
